# Melatonin and Its Protective Role against Biotic Stress Impacts on Plants

**DOI:** 10.3390/biom10010054

**Published:** 2019-12-28

**Authors:** Mohamed Moustafa-Farag, Abdulwareth Almoneafy, Ahmed Mahmoud, Amr Elkelish, Marino B. Arnao, Linfeng Li, Shaoying Ai

**Affiliations:** 1 Institute of Agricultural Resources and Environment, Guangdong Academy of Agricultural Sciences, Guangzhou 510640, Guangdong, China; 2 Horticulture Research Institute, Agriculture Research Center, 9 Gmaa St, Giza 12619, Egypt; 3 Department of Biology sciences, College of Education and Science at Rada’a, Albaydaa University, Rada’a, Yemen; 4 Laboratory of Germplasm Innovation and Molecular Breeding, Institute of Vegetable Science, Zhejiang University, Hangzhou 310058, Zhejiang, China; 5 Botany Department, Faculty of Science, Suez Canal University, Ismailia 41522, Egypt; 6 Department of Plant Physiology, Faculty of Biology, University of Murcia, 30100 Murcia, Spain

**Keywords:** melatonin, plant hormone, biotic stress, bacteria, fungi, virus, antioxidants

## Abstract

Biotic stress causes immense damage to agricultural products worldwide and raises the risk of hunger in many areas. Plants themselves tolerate biotic stresses via several pathways, including pathogen-associated molecular patterns (PAMPs), which trigger immunity and plant resistance (R) proteins. On the other hand, humans use several non-ecofriendly methods to control biotic stresses, such as chemical applications. Compared with chemical control, melatonin is an ecofriendly compound that is an economical alternative strategy which can be used to protect animals and plants from attacks via pathogens. In plants, the bactericidal capacity of melatonin was verified against *Mycobacterium tuberculosis*, as well as multidrug-resistant Gram-negative and -positive bacteria under in vitro conditions. Regarding plant–bacteria interaction, melatonin has presented effective antibacterial activities against phytobacterial pathogens. In plant–fungi interaction models, melatonin was found to play a key role in plant resistance to *Botrytis cinerea*, to increase fungicide susceptibility, and to reduce the stress tolerance of *Phytophthora infestans*. In plant–virus interaction models, melatonin not only efficiently eradicated apple stem grooving virus (ASGV) from apple shoots in vitro (making it useful for the production of virus-free plants) but also reduced tobacco mosaic virus (TMV) viral RNA and virus concentration in infected *Nicotiana glutinosa* and *Solanum lycopersicum* seedlings. Indeed, melatonin has unique advantages in plant growth regulation and increasing plant resistance effectiveness against different forms of biotic and abiotic stress. Although considerable work has been done regarding the role of melatonin in plant tolerance to abiotic stresses, its role in biotic stress remains unclear and requires clarification. In our review, we summarize the work that has been accomplished so far; highlight melatonin’s function in plant tolerance to pathogens such as bacteria, viruses, and fungi; and determine the direction required for future studies on this topic.

## 1. Introduction

Biotic stress can be described as damage caused to crops by several live organisms such as fungi, bacteria, viruses, parasitic nematodes, insects, weeds, and other indigenous or grown plants. The severity of these effects and the resulting loss of crops depend on multiple factors, such as causal organisms, environmental conditions, and corresponding levels of crops and causal organisms [1]. Pathogens such as fungi, bacteria, nematodes, and viruses are mainly accountable for plant diseases. Among other symptoms, fungi and bacteria may cause leaf spots, vascular wilts, and cankers and can impact various plant organs. Nematodes take up plant cell content and attack all plant parts; they are also able to ease the entry of soil-borne pathogens into the root system, causing nutrient-deficiency-related symptoms such as stunting or wilting. Viruses not only produce local lesions but also cause systemic damage, resulting in malformations, stunting, and chlorosis in various plant sections, even if their hosts are often not killed [2]. Insects and mites, on the other hand, should be emphasized. They cause damage to plants by laying eggs or feeding. Piercing–sucking insects can function as vectors of viruses and deliver them to plants through their own stylets [3].

## 2. How Plants Defend Themselves against Biotic Stresses

Plants usually have a sophisticated immune system to cope with biotic stresses. First, plants have physical obstacles, such as waxes, dense cuticles, and special trichomes, which stop pathogens or insects from settling on plants. Furthermore, plants generate chemical complexes to protect themselves against herbivores and pathogens [4]. Additionally, plants can recognize pathogens via two pathways that activate defense responses. The first one is the pattern recognition receptor, which detects pathogen-associated molecular patterns (PAMPs), such as flagellin, bacterial lipopolysaccharides, fungal chitin, peptidoglycans, and quorum sensing. This basic form of defense is known as PAMP-triggered immunity (PTI) [5]. The second pathway of the immune system is plant resistance (R) proteins, which recognize pests’ or pathogens’ specific effectors (Avr proteins) and activate the plant defense response through a process known as effector-triggered immunity (ETI). This kind of mechanism can activate hypersensitive responses (HR), which include programming of cell death in impacted cells and its surrounding areas [6]. Within signaling pathways induced by PTI and ETI, several plant hormones stand out: ethylene (ET), jasmonic acid (JA), and salicylic acid (SA). While the SA mechanism promotes resistance defense to hemi-biotrophic and biotrophic pathogens, the ET and JA pathways are commonly induced against chewing insects and necrotrophic pathogens in *Arabidopsis* [7]. Generally, three hormones of SA, JA, and ET act as signaling molecules of two effective defense mechanisms against plant pathogens. The first one is called systemic acquired resistance (SAR), which is activated after primary infection with a necrotizing pathogen and is accompanied by increasing concentrations of SA and related proteins of pathogenesis [8]. The second one is called induced systemic resistance (ISR), which is a kind of plant resistance that is activated via specific strains of non-pathogenic root-colonizing bacteria; its signaling requires JA and ET [9]. Likewise, phytophagous insects cause plants to exhale volatiles to attract their enemies and alert their neighbor plants to imminent threats by recognition of conserved herbivore-associated elicitors of the invading insect [10].

## 3. Management Approaches of Biotic Stress

Although plants have several defense mechanisms to tolerate and adapt to stress conditions, biotic stresses cause massive economic losses from important crops every year. The direct yield loss due to these pests ranges between 20% and 40% of agricultural productivity worldwide [11,12,13,14,15].

Different control methods have been applied to prevent or mitigate biotic stress initiated by agricultural pests, including chemical, genetic, biological, and agricultural controls, as well as integrated pest management, which incorporates the use of different pest control approaches and can alter the suitability of pests’ microenvironment and influence their prevalence [16]. Agricultural producers prefer to use chemical control, as it is an effective management option for many plant pests. However, the development of pathogens that are resistant to these chemicals and the effects on untargeted organisms are major environmental concerns [17]. In addition, the high expense of using these chemicals in agricultural production systems and the great impact of their residues on human health and the environment have prompted the search for control approaches that are less toxic to non-target organisms, renewable, highly biodegradable, and more economical than synthetic chemical pesticides [18].

## 4. Melatonin as an Alleviating Agent against Plant Pathogens

Melatonin (*N*-acetyl-5-methoxytryptamine), an animal hormone was discovered in the pineal gland of the cow in 1958 [19], has multiple functions in humans and animals, and it has been seen since its discovery in plants in 1995 [20,21] to present a multitude of regulatory functions also in plants [22,23,24]. Melatonin acts as an excellent antioxidant against reactive oxygen and nitrogen species (ROS/RNS). One of the most studied functions of melatonin in plants is its role as a protective agent against various stress situations [25]. In addition to acting as an antioxidant agent, melatonin induces numerous changes in gene expression. These regulatory changes are beneficial for dealing with adverse situations, assuming reinforcement against plant stress. Thus, melatonin reinforces physiological processes such as germination, photosynthesis, stomatic uptake, growth, rooting, osmoregulation, anti-senescence, primary and secondary metabolism, and also, plant hormone regulation [24,26].

Melatonin is synthesized in plants through a route from tryptophan, although it differs in several of its stages and enzymes from biosynthesis in animals [27,28]. In their mini review, Dhole, et al. [29] reported that melatonin was synthesized in plant in various subcellular sites with intensive enzymatic support of tryptophan hydroxylase, tryptamine 5-hydroxylase, caffeic acid *O*-methyltransferase, *N*-acetylserotonin methyltransferase, tryptophan decarboxylase and serotonin *N*-acetyltransferase enzymes. Phytomelatonin synthesis starts from tryptophan decarboxylase which catalyzes tryptophan into 5-hydroxytryptophan or tryptamine to serotonin. The next step in the biosynthesis of melatonin is the catalyzed via tryptophan 5-hydroxylase and is associated-hydroxylation reaction, which is mainly moderated in plants by cytochrome P450-dependent monooxygenases (P450s) and 2-oxoglutarate-dependent dioxygenases (2-ODDs). Additionally, this also catalyses *N*-Acetyl tryptamine to *N*-acetyl serotonin reactions and tryptophan to 5-hydroxytryptophan. The second-to-last step in this pathway is catalyzed by serotonin *N*-acetyltransferase (SNAT), which catalyzes the carrying of the acetyl group from acetylcoenzyme A to the array of aminoglycosides and arylalkylamines molecules. The last step is 5-hydroxyindol *O*-methyltranseferase which catalyzes *N*-acetylserotonin into phytomelatonin through an *O*-methyltransferase (OMT) reaction. More information about this pathway can be found in Kaur, et al. Ref. [30].

Considering that melatonin is an environmentally friendly molecule, it may represent an economical alternative strategy to induce plant protection against biotic stress. Many studies have shown that melatonin can play a key role in plant protection against biotic stress. melatonin has been reported to have immunomodulatory, antioxidant, anti-inflammatory, and neuroprotective activities in animals [31,32,33,34], so it can be applied as an effective therapeutic substitute for the suppression of microbial diseases. On the other hand, recently, many important findings have shown the beneficial effect of melatonin in plant–pathogen interaction. In this regard, further related details are extensively discussed in the following subsections.

### 4.1. Melatonin as an Antiviral Agent

**Figure 1:**
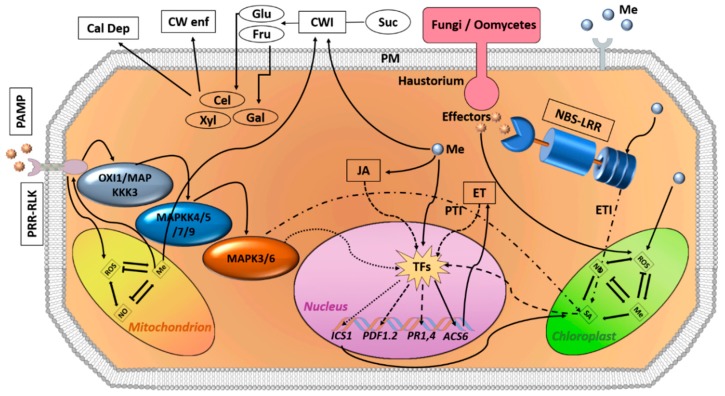
Expected model of melatonin-related defense pathways according to the findings obtained from many studies [24,25,39,40,41,42,43,44,45,46,47,48,49,50,51]. Abbreviations: NBS-LRR, nucleotide-binding site leucine-rich repeat; LRR-RLKs, leucine-rich repeat receptor-like kinases; ETI, effector triggered immunity; PTI, PAMP-triggered immunity; TFs, transcription factors; CWI, cell wall invertase; CW enf, cell wall enforcement; Cal Dep, callose deposition; PM, plasma membrane; Suc, sucrose; Glu, glucose; Fru, fructose; Cel, cellulose; Gal, galactose; Xyl, xylose; ROS, reactive oxygen species; NO, nitric oxide; PAMP, pathogen-associated molecular pattern; SA, salicylic acid; JA, jasmonic acid; ET, ethylene; MAPK, mitogen-activated protein kinase. Lined and dotted arrows denote the assumed mechanisms; lines with bars point to negative action in the respective pathway.

In animals, the antiviral activity of melatonin has been demonstrated in many investigations. For example, melatonin treatment reduced the deleterious effects of ROS involved in the dissemination of the Venezuelan equine encephalomyelitis (VEE) virus. Melatonin administration significantly decreased blood and brain viruses in comparison with infected control mice [31]. In another study, melatonin and the antiviral drug ribavirin cotreatment significantly increased the survival rate of influenza-virus-infected mice compared with ribavirin treatment only [35]. In this context, melatonin could regulate the autophagy process during some viral infections due to its high antioxidation efficiency and its ability to suppress endoplasmic reticulum stress [36]. Until now, few studies have considered the antiviral effect of melatonin in plants. Regarding this, treatment with exogenous melatonin (100 µM, twice) resulted in the reduction of tobacco mosaic virus (TMV) viral RNA and virus concentration in infected *Nicotiana glutinosa* and *Solanum lycopersicum* seedlings. This positive effect of melatonin was attributed to the increase of SA concentrations in the NO-dependent pathway [37]. Moreover, melatonin efficiently eradicated apple stem grooving virus (ASGV) from the in vitro virus-infected apple shoots of “Gala” and could be a useful means to produce virus-free plants [38]. The beneficial antiviral activities of melatonin in plants are shown in Table 1 and Figure 1. Further studies are required to elucidate melatonin’s effect on plant–virus interaction.

### 4.2. Melatonin Bioactivity against Bacteria

Melatonin defensive activities against bacterial infections in animals have been tested under in vitro and in vivo conditions. The bactericidal capacity of melatonin was verified against multidrug-resistant Gram-negative and -positive bacteria, such as carbapenem-resistant *Pseudomonas aeruginosa*, *Acinetobacter baumannii*, and methicillin-resistant *Staphylococcus aureus* under in vitro conditions [52]. Also, the strong inhibitory action of melatonin against *Mycobacterium tuberculosis* (H37Rv strain) was revealed after application of a 10 µM concentration; however, the combined application of melatonin with isoniazid inhibited the growth of bacteria three to four times more than any compound alone [53]. Besides its ability to potentiate immune responses, additional explanations have been provided to clarify the mechanism of melatonin’s antibacterial action in animals, including cellular cAMP and Ca^2+^ regulation, reduction of intracellular substrates, free radical formation, and binding to the bacterial cell wall, which causes cytoderm destabilization [32,33,34,35,36,37,38,52,53,54]. In plant–bacteria interaction, melatonin has presented effective antibacterial activities against phytobacterial pathogens. For instance, incidence of rice bacterial leaf streak (BLS) due to *Xanthomonas oryzae* pv. *oryzicola* (*Xoo*) was reduced by exogenous melatonin treatment on leaves by 17% [55]. Additionally, the direct inhibitory effect of melatonin was reported on phytopathogenic bacteria such as *X. oryzae* pv. *oryzae* and *X. oryzae* pv. *oryzicola* [55,56]. Likewise, Nehela, et al. [57] found that supplemented melatonin treatment could invert the negative disorders of *Candidatus* Liberibacter asiaticus (*C*Las) (the causal agent of citrus greening disease) on its insect vector by enhancement of melatonin content, extend the longevity of healthy and infected vectors, and reduce the *C*Las bacterial population within the vector psyllids. In another study, it was reported that direct transcriptional activators of melatonin biosynthesis genes in cassava crops, namely, *MeRAV2 and MeRAV1* genes, are required to confer plant disease resistance against bacterial blight of cassava [58]. Antibacterial mechanisms against plant pathogenic bacteria are characterized by upregulation of defense genes such as *plant defensin 1.2* (*PDF1.2*), *plant resistance 1* (*PR1*), and *PR5* through several signal transduction pathways, such as augmentation of NO levels in plants, which collaborate with melatonin in upregulating SA-associated genes [43] such as *PAD4*, *EDS1*, *PR5*, *PR1*, and *PR2*. Also, melatonin can stimulate mitogen-activated protein kinase (MAPK) cascades in (Pst)-DC3000-infected *Arabidopsis thaliana*, which in turn upregulate SA biosynthesis gene *isochorismate synthase 1* (*ICS1*) [44,47,48]. In *A. thaliana* plants infected with (Pst) DC3000, Zhao, et al. [45] found that the high cell wall invertase (CWI) activity within melatonin-treated *Arabidopsis* leads to improved cell wall strengthening and callose-depositing factors (cellulose, xylose, and galactose). Additionally, sugar and glycerol are involved in melatonin-related defense toward (Pst) DC3000 in SA- and NO-dependent pathways in *Arabidopsis*. Melatonin therapy is reported to induce 1-aminocyclopropane-1-carboxylate synthase 6 (ACS6), which is the main enzyme in ethylene biosynthesis, which in turn induces *PDF1.2* expression [44]. Since JA can also induce *PDF1.2* expression, we cannot exclude JA’s potential involvement with melatonin in pathogen resistance [44,45,46].

Also, Qian, et al. [42] reported on the participation of sugar and glycerol in melatonin-associated protection against (Pst) DC3000 through SA- and NO-dependent pathways in *Arabidopsis* [42]. Recently, exogenous melatonin treatment was found to activate ETI- and PTI-associated genes in watermelon and *Arabidopsis* according to transcriptomic data [40,41]. All of the bactericidal activities of melatonin, the related mechanisms involved, and the resulting constructive effects in plants are summarized in Table 2 and Figure 1.

### 4.3. Antifungal Effect of Melatonin

Concerning animal models, melatonin was shown to have therapeutic advantages due to its immune regulatory role in *Candida* sepsis and classic antimycotic therapy, where it was able to lower interleukin-6 levels and reduce the time needed to improve *Candida* sepsis in rats [59]. Furthermore, results from some investigations support the hypothesis that melatonin increases phagocytic activity and decreases oxidative stress during candidiasis [60,61,62]. In a plant–fungi interaction model, melatonin played a promotional role in the resistance of tomato fruit to *Botrytis cinerea* via the regulation of H_2_O_2_ generation and the jasmonic acid signaling mechanism [63]. Melatonin can increase fungicide susceptibility, enhance vulnerability of *Phytophthora infestans* to different environmental stress, decrease the dosage level, and promote the efficiency of fungicide treatment against potato late blight [64]. An increase of melatonin accumulation in plants produces more resistance against foliar pathogens such as powdery mildew and soil-borne oomycetes in watermelon and other cucurbits through changes in the expression of the genes associated with ETI- and PAMP-mediated defenses [40]. After melatonin treatment, both the incidence of *A. thaliana* infection by *Plasmodiophora brassicae* and the number of pathogen sporangia were reduced, and this reduction was attributed to the high expression of the JA-responsive *PR3* and *PR4* genes [65]. Powdery mildew on cucumber plants decreased significantly after application of 100 μM of melatonin. This pretreatment reduced the disease index by activating antioxidant enzymes and the expression of antioxidant-related genes [66]. Zhang, et al. [67] demonstrated that the combined application of melatonin and ethylicin (an oomycete antifungal) has a synergistic effect that inhibits the in vitro and in vivo growth of *Phytophthora nicotianae* through disturbed amino acid metabolic homeostasis of this fungus. Exogenous application of melatonin in replant soil enhances apple seedling growth, raises K levels, and stimulates photosynthesis, which subsequently relieves the disorders of replant disease [68]. Similar results have been obtained in fungi such as *Botrytis* spp., *Penicillium* spp., *Fusarium* spp., *P. nicotianae*, and *Alternaria* spp. [67,68,69]. Furthermore, several investigations have studied the role of endophytic rhizobacteria in the reinforcement ability of plants to produce melatonin [70,71]. In addition, melatonin inhibited the total microorganisms, including mold, yeast, and bacteria, in stored apple juice during the storage time (4–12 h) [72]. Unlike the other studies mentioned, melatonin was found to reduce resistance to green mold disease on the fruit of citrus caused by *Penicillium digitatum* through scavenging of defense-related ROS in the infected fruits [73]. Several interpretations to explain the protective role of melatonin against plant fungal pathogens have been proposed. For instance, some researchers have ascribed the defense mechanism of melatonin to its ability to maintain H_2_O_2_ cellular concentration and the generation and regulation of antioxidant enzyme activities [50,51].

Recently, exogenous melatonin treatment was found to activate ETI- and PTI-associated genes in watermelon and *Arabidopsis* according to transcriptomic data [40,41]. In addition, melatonin plays a key role in the regulation of ROS and reactive nitrogen species (RNS) rates in plants, which act as a signal in many cellular and physiologic responses to both abiotic and biotic stresses, both directly (as scavengers of ROS/RNS) and indirectly (as gene regulators of the redox network) [25,49]. Other melatonin defense mechanisms are elaborated in Table 3 and Figure 1.

## 5. Conclusions and Research Gaps

Recently, melatonin has gained a considerable amount of interest because of its unique advantages in plant growth regulation and increasing plant resistance effectiveness against different forms of biotic and abiotic stresses. In that respect and in light of recent studies, there has been great progress in exploring the different roles of melatonin in plants, and several attempts have been made to clarify the positive effect of this molecule on plants. Although many regulatory elements of the melatonin-related defense signaling network have been investigated, clarification remains necessary for some upstream components in this network [42,43,44,47]. Recently, this knowledge gap was partially bridged when *AtPMTR1*, the first receptor for phytomelatonin, was discovered in *Arabidopsis* [75]. This finding has created new expectations for melatonin’s function as a plant hormone [49]. Although this important discovery has elucidated the upstream pathway regarding stomata closure phenotype, there is still the need to explore the crucial role of other melatonin receptor-mediated signaling pathways during biotic/abiotic stress in plants [25,76], although interesting transcriptome approaches have already been made [41]. Recent studies that discerned the ability of melatonin to upregulate ETI and PTI defense-related genes need to affirm their findings within proteomic approaches by establishing a clear signal transduction pathway for this process. Plant growth promotion rhizobacteria (PGPR) have been extensively studied due to their beneficial role in shielding plants against pathogenic diseases and enhancing plant development and growth through nitrogen fixation, siderophore production, phosphorus solubilization, and increasing plant hormone accumulations [77,78,79]. Many studies have reported that PGPR can produce melatonin and subsequently raise its endogenous levels in different plant organs [70,71,80]. However, it is necessary to investigate in detail the effect of combined applications of melatonin and PGPR on plant defense against several environmental stressors. Also, the synergistic effect of combined melatonin and antifungal treatments is an interesting way by which to obtain a high degree of plant pathogen resistance response with lower concentrations of chemical fungicides. Furthermore, insufficient evidence is available regarding the impact of melatonin on plant infection by viruses, nematodes, and insects, so further research in this respect is required.

## Figures and Tables

**Table 1:** Effect of melatonin on plant–virus pathosystem.

Plant Name	Pathogen Name	Melatonin Dosage (µM)	Delivery Method	Involved Mechanism	Resulting Effect	Ref.
*Nicotiana glutinosa* and *Solanum lycopersicum*	Tobacco mosaic virus (TMV)	100 (twice)	Root irrigation	Salicylic acid (SA) concentrations lead to increased expression of *PR1* and *PR5* genes	Reduction of virus concentration in infected plants	[37]
*Malus domestica*	Apple stem grooving virus (ASGV)	15	To the shoot proliferation medium	Not reconnoitered	Eradication of virus from previously infected shoot tips	[38]

SA = Salicylic acid; *PR1* and *PR5* = plant resistance genes 1 and 5.

**Table 2:** Beneficial action of melatonin in plants infected with bacterial pathogens.

Plant	Pathogen	Melatonin Dosage (µM)	Delivery Method	Mechanism	Effect	Ref.
*Arabidopsis thaliana*, *Nicotiana benthamiana*	*Pseudomonas syringae*	1 or 10	Leaf treatment	▲ Expression of defense genes *PR1*, *PR5*, and *PDF1.2*	Inhibition of pathogen propagation	[44]
*A. thaliana*	*P. syringae*	10	Leaf treatment	▲ Pathogenesis-related (PR) genes by the harmonizing signaling between SA and ET	Increase resistance against pathogen	[39]
*A. thaliana*, *N. benthamiana*	*P. syringae*	1	Leaf treatment	▲ Induction of PR genes through MAPK signaling cascades	Disease resistance	[47]
*A. thaliana*	*P. syringae*	20	Added to nutrient solution	Involvement of sugars and glycerol in melatonin-mediated innate immunity in SA- and NO-dependent pathways	Disease resistance	[42]
*A. thaliana*	*P. syringae*	20	Added to nutrient solution	▲ NO and melatonin levels in leaves ▲ Defense-related genes	Improvement of disease resistance	[43]
*A. thaliana*	*P. syringae*	50	Added to plant culture medium	▲ Activities of CWI and vacuolar invertase (VI)	Cell-wall reinforcement and callose deposition during infection	[45]
*A. thaliana*	*P. syringae*	1	Leaf treatment	Induction of *PR1* and *ICS1* expression genes through MAPK cascades in coexistence H_2_O_2_ and NO	Disease resistance	[48]

▲ = increasing in content or action; SA = salicylic acid; *PR1* and *PR5* = plant resistance genes 1 and 5; ET = ethylene; MAPK = mitogen-activated protein kinase; NO = nitric oxide; *PDF1.2* = plant defensin 1.2 gene; *ICS1* = isochorismate synthase 1 gene; H_2_O_2 =_ hydrogen peroxide.

**Table 3:** Role of exogenous melatonin treatment in plant–fungal interaction.

Plant Name	Pathogen Name	Melatonin Dosage (µM)	Delivery Method	Involved Mechanism	Resulting Effect	Ref.
*Malus prunifolia*	*Diplocarpon mali*	50–500	Root irrigation	Maintain intracellular H_2_O_2_ concentrations ▲ Activities of plant defense-related enzymes	Alleviating disease damage Fungal infection resistance Lesion reduction	[51]
*Musa acuminata*	*Fusarium oxysporum*	100	Leaf and root treatment	▲ Resistance induced via regulating the expression of *MaHSP90s* gene	Improvement of disease resistance	[74]
*Fragaria ananassa*	*Botrytis cinerea* and *Rhizopus stolonifer*	100	Fruit dipping	▲ H_2_O_2_ levels ▲ Antioxidant enzyme activities	Reduction of postharvest decay in stored strawberry fruits	[50]
*Citrullus lanatus*	*Podosphaera xanthii* and *Phythophthora capsici*	100	Leaf treatment	▲ Upregulation of PTI- and ETI-associated genes	Disease resistance	[40]
*A. thaliana*	--------	1 × 10^−4^ or 100	Seedling rinsing	▲ Upregulation of genes involved in abscisic acid (ABA), ET, SA, and JA pathways	Increase plant resilience to biotic and abiotic stress.	[41]

▲ = increasing in content or action; H_2_O_2 =_ hydrogen peroxide; SA = salicylic acid; ET = ethylene; PTI = pattern-triggered immunity; ETI = effector-triggered. immunity; JA = jasmonic acid; ABA = abscisic acid.
